# Effects of *APOE* ε2 allele on basal forebrain functional connectivity in mild cognitive impairment

**DOI:** 10.1111/cns.14038

**Published:** 2022-12-05

**Authors:** Xiaocao Liu, Qingze Zeng, Xiao Luo, Kaicheng Li, Xiaopei Xu, Luwei Hong, Jixuan Li, Xiaojun Guan, Xiaojun Xu, Peiyu Huang, Min‐Ming Zhang, the Alzheimer's Disease Neuroimaging Initiative (ADNI)

**Affiliations:** ^1^ Department of Radiology The 2nd Affiliated Hospital of Zhejiang University School of Medicine Hangzhou China

**Keywords:** Alzheimer's disease, *APOE*, basal forebrain, functional connectivity, mild cognitive impairment

## Abstract

**Background:**

Basal forebrain cholinergic system (BFCS) dysfunction is associated with cognitive decline in Alzheimer's disease (AD) and mild cognitive impairment (MCI). Apolipoprotein E (*APOE*) ε2 is a protective genetic factor in AD and MCI, and cholinergic sprouting depends on *APOE*.

**Objective:**

We investigated the effect of the *APOE* ε2 allele on BFCS functional connectivity (FC) in cognitively normal (CN) subjects and MCI patients.

**Method:**

We included 60 MCI patients with *APOE* ε3/ε3, 18 MCI patients with *APOE* ε2/ε3, 73 CN subjects with *APOE* ε3/ε3, and 36 CN subjects with *APOE* ε2/ε3 genotypes who had resting‐state functional magnetic resonance imaging data from the Alzheimer's disease Neuroimaging Initiative. We used BFCS subregions (Ch1‐3 and Ch4) as seeds and calculated the FC with other brain areas. Using a mixed‐effect analysis, we explored the interaction effects of *APOE* ε2 allele × cognitive status on BFCS‐FC. Furthermore, we examined the relationships between imaging metrics, cognitive abilities, and AD pathology markers, controlling for sex, age, and education as covariates.

**Results:**

An interaction effect on functional connectivity was found between the right Ch4 (RCh4) and left insula (*p* < 0.05, corrected), and between the RCh4 and left Rolandic operculum (*p* < 0.05, corrected). Among all subjects and *APOE* ε2 carriers, RCh4‐left Insula FC was associated with early tau deposition. Furthermore, no correlation was found between imaging metrics and amyloid burden. Among all subjects and *APOE* ε2 carriers, FC metrics were associated with cognitive performance.

**Conclusion:**

The *APOE* ε2 genotype may play a protective role during BFCS degeneration in MCI.

## Graphical abstract

This article showed the evidence that the APOE ε2 allele impacts the FC patterns of basal forebrain cholinergic system (BFCS) subregions in MCI, which may be one of the important mechanisms contributing to its protective role. Furthermore, the APOE ε2 allele is associated with lower Tau deposition in BFCS subregions. The article might provide original insights for understanding the possible protection of APOE ε2 alleles in MCI patients.

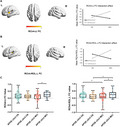

## 1 INTRODUCTION

Alzheimer's disease (AD) is the most common cause of dementia and is clinically characterized by progressive and irreversible cognitive decline. Extracellular β‐amyloid (Aβ) deposition and intraneuronal neurofibrillary tangles are the major neuropathological alterations.^1^ Numerous factors lead to the onset of AD, and apolipoprotein E (*APOE*, gene) is one of the most vital genetic factors for sporadic AD.^2^, ^3^, ^4^ The *APOE* ε4 allele is associated with an increased risk of AD.^5^, ^6^, ^7^ Conversely, the ε2 allele of *APOE* has a protective effect against AD.^6^, ^8^

Accumulated evidence has supported that the basal forebrain cholinergic system (BFCS) degenerates in mild cognitive impairment (MCI) and AD.^9^, ^10^, ^11^, ^12^ According to previous studies,^13^, ^14^ the BFCS regions can be defined as four subregions, Ch1‐Ch4, among which the cholinergic component of the nucleus basalis of Meynert (nbM) is designated as Ch4. Previous structural magnetic resonance imaging (MRI) and fMRI studies have also reported atrophy and disruption of intrinsic activity in the BFCS and BFCS subregions in both AD and MCI patients.^15^, ^16^, ^17^, ^18^, ^19^, ^20^ In the MCI stage, the pathological deposition, especially tau, impairs cholinergic functions of the BFCS.^21^ Furthermore, it is plausible that abnormal functional connectivity (FC) of the BFCS with other brain regions might contribute to the cognitive decline, including episodic memory, executive function, and information processing, in MCI.^22^, ^23^, ^24^ Additionally, cholinergic sprouting depends on *APOE*, the principal cholesterol and lipid transport protein for neurons required for sprouting.^25^ A recent study showed that the *APOE* genotype may influence compensatory cholinergic mechanisms.^26^ More specifically, the *APOE* ε4 allele is associated with deficits in cholinergic hippocampal compensatory sprouting in response to cholinergic deafferentation.^27^ Thus, we hypothesized that *APOE* ε2 might be related to the reduced the BFCS degeneration.

Resting‐state functional MRI (rs‐fMRI) is regarded as a sensitive biomarker for both MCI and AD. It can detect the abnormalities representing functional impairment even earlier than structural MRI.^28^, ^29^ Substantial functional MRI studies have confirmed that FC disruption alters in the early stage of the disease^30^ and provided complementary information on BFCS function.^18^, ^23^, ^24^ Some studies have also reported differences in the effects of the *APOE* ε2 allele under various cognitive statuses. For instance, compared with *APOE* ε3 and ε4 carriers, ε2 carriers with MCI show different higher FC between the right entorhinal cortex (ERC) and right precuneus (PCUN), while healthy control ε2 carriers have lower FC between those two structures. Another study found that cognitively normal (CN) subjects and MCI patients carrying *APOE* ε2 allele showed different alterations in the default mode network (DMN).^31^ However, how *APOE* genotypes, and especially the *APOE* ε2 allele, affect FC alterations of the BFCS subregions in MCI patients remains unknown. Moreover, the effect of the *APOE* ε2 allele on different diagnoses (MCI and CN subjects) is worthy of attention.

Previous studies have revealed that *APOE* ε2 carriers were associated with less Aβ plaques accumulation,^32^ more effective Aβ clearance,^33^, ^34^ and milder Braak neurofibrillary tangles (NFT) stages.^35^ Thus, we aimed to investigate the effect of the *APOE* ε2 allele on BFCS‐FC in CN subjects and MCI patients. Considering the protective role of *APOE* ε2, we hypothesized that *APOE* ε2 allele carriers might show lower pathology deposition and maintain stable cognition during the MCI stage via functional alterations between the BFCS and other cerebral regions.

## 2 METHODS AND MATERIALS

### 2.1 Study participants

All data used in this study were obtained from the Alzheimer's Disease Neuroimaging Initiative (ADNI) database (http://www.loni.ucla.edu/ADNI). We identified nondemented subjects (characterized as either CN or MCI) from the ADNI GO/2/3 databases (the flowchart is presented in Figure S1). All subjects had undergone rs‐fMRI scans, neuropsychological evaluations, and *APOE* gene assessment.

The criteria for MCI in the ADNI protocol were (1) subjective memory complaints; (2) objective memory loss, which was defined as scoring below an education‐adjusted cutoff score for delayed recall on the Wechsler Memory Scale‐Logical Memory (WMS‐LM) test; (3) a global Clinical Dementia Rating (CDR) score of 0.5; (4) a Mini‐mental State Examination (MMSE) score ≥ 24; (5) the on‐site physician could not diagnose dementia at the time of screening; (6) no signs of depression (geriatric depression scale, GDS score <5). Furthermore, the criteria for CN were: (1) an MMSE score ≥ 24; (2) CDR score = 0; (3) no report of any cognition complaint; (4) GDS score <5. The exclusion criteria were (1) serious medical, neurological, or psychiatric illness, (2) history of head trauma, (3) history of using non‐AD‐related medication known to influence cerebral function, and (4) drug or alcohol abuse. Additionally, participants with the ε2/ε4 genotype were excluded because the ε2 and ε4 alleles have opposing effects on AD.^36^ Furthermore, we did not identify *APOE* ε2 homozygous participants who met the inclusion requirements.

We included 54 *APOE* ε2/ε3 carriers, including 18 MCI patients and 36 CN subjects. Subsequently, we included 133 *APOE* ε3/ε3 carriers who were matched for sex, age, and education level to *APOE* ε2/ε3 carriers. Imaging data and demographic information were obtained from the ADNI database before April 1, 2021. We also downloaded the standardized uptake value ratio (SUVR) of [18F] AV1451 positron emission tomography (Tau PET) and [18F] AV45 PET (Aβ PET) from the ADNI database. The time interval between MRI and PET scans was <12 months. PET data were not available for all subjects in the current study. Specifically, of 187 subjects, 100 had [18F] AV1451 PET, and 124 had [18F] AV45 PET. And we compared the difference among the subjects who had PET data instead of replacing missing data with an inserted value.

### 2.2 Neuropsychological assessment

Several neuropsychological tests were used in this study, including the Montreal Cognitive Assessment (MOCA) for global cognitive scores. Additionally, neuropsychological assessments for different cognitive domains, such as memory function (WMS‐LM, immediate and delayed recall), attention function (Trail‐making Test part A, TMT‐A), execution function (Trail‐making test, Part B, TMT‐B), language ability (Boston Naming Test, BNT), and visual–spatial function (Clock Drawing Test, CDT) were included.

### 2.3 APOE genotyping

*APOE* genotyping was performed as previously described.^37^ Specifically, DNA was extracted from peripheral blood cells. Then, the cells were sent via overnight delivery to the University of Pennsylvania AD Biofluid Bank Laboratory (*ApoE*‐Results, ADNI 1, GO, 2, 3) for analysis.

### 2.4 MRI acquisition

All subjects were scanned using 3.0‐Tesla MRI scanners. The parameters used for the rs‐fMRI echo‐planar imaging sequence were as follows: TE = 30 ms; TR = 3000 ms; slice number = 48; slice thickness = 3.3 mm; flip angle = 90°, and matrix = 64 × 64. In accordance with the human scanning protocol of the ADNI database protocol (http://adni.loni.usc.edu/methods/documents/), all participants kept their eyes open during the entire rs‐fMRI scan.

### 2.5 PET acquisition

The [18F] AV45 and [18F] AV1451 PET scans were acquired using various PET scanners (Siemens, GE, and Philips). The data acquisition procedures can be found at http://adni.loni.usc.edu/methods/documents/. In brief, the PET scan consisted of 4 × 300‐s frames measured 50 min after injection of 370 MBq (10 ± 1.0 mCi) of [18F]‐florbetapir AV‐45 or [18F]‐flortaucipir AV1451.

### 2.6 PET image analysis

The [18F] florbetapir PET (UCBERKELEYAV45_01_14_21) and [18F] flortaucipir PET (UCBERKELEYAV1451_01_14_21) results were processed by UC Berkeley and Lawrence Berkeley National Laboratory. Briefly, T1 images were processed using Freesurfer v5.3, and [18F] florbetapir and [18F] flortaucipir images were co‐registered to native T1 maps using Statistical Parametric Mapping 12 (spm12; http://www.fil.ion.ucl.ac.uk/spm/). Standardized uptake value ratio (SUVR) images were created using a gray reference region of the cerebellum, and images were normalized to Montreal Neurological Institute (MNI) space using the parameters from the co‐registered T1 maps. The florbetapir SUVR and flortaucipir SUVR values were used to measure Aβ and tau pathology in subjects separately. Specifically, the flortaucipir SUVRs of Braak stage I–II, Braak stage III–IV, and Braak stage V–VI were used to measure tau pathology. Participants' Aβ burden was estimated using a summary whole cerebrum SUVR.

### 2.7 Preprocessing of fMRI data

The rs‐fMRI data were pre‐processed by the DPABI toolbox^38^ with SPM12 on the MATLAB platform (MathWorks, Natick, MA, USA). We discarded the first 10 time points of the rs‐fMRI data because of the instability of the initial MRI signal and the subjects' adaptation to the scanning noise. Then the remaining 130 images were corrected for both timing differences between each slice and head motion (six‐parameters rigid body). Subjects with more than 2.0 mm maximum displacement in the *x*, *y*, or *z* direction or 2.0° of angular motion during the whole scan were excluded. Twenty‐four participants were excluded based on excessive head motion. Subsequently, the fMRI data were warped to the MNI space using the EPI template^39^ and then resampled into 3 × 3 × 3 mm^3^ cubic voxels. Finally, the fMRI data were smoothed using a 6 mm full width at half maximum kernel. To minimize physiological noise, the Friston 24 head motion parameters, white matter (WM) signal, and cerebrospinal fluid (CSF) signal were corrected as nuisances. Then, the rs‐fMRI images were bandpass‐filtered at 0.01 and 0.08 Hz to reduce the effect of low‐frequency drifts and high‐frequency physiological noise.

### 2.8 Seed‐based FC analysis

#### 2.8.1 Cholinergic basal forebrain regions of interest

The masks of the regions of interest (ROIs) (bilateral Ch1‐3 and Ch 4) were extracted from probabilistic cytoarchitectonic maps using the SPM Anatomy Toolbox v22c for ROI‐based FC analysis^40^ (Figure S2). Furthermore, to avoid signal loss or mixed with WM, CSF, each seed was overlapped in both fMRI and T1 images for examination.

#### 2.8.2 FC analysis

The voxel time courses of the ROIs were extracted, and then the FC of Ch1‐3 and Ch4 was calculated using Dynamic Brain Connectome (Dynamic BC) toolbox^41^ (V2.0 http://restfmri.net/forum/DynamicBC). Specifically, each mask was resampled to the dimension of our normalized functional image with a 3 × 3 × 3 voxel size for seed‐based resting‐state FC analyses. Next, the resting‐state FC maps were generated by calculating the Pearson correlation between the time course of the ROIs and the whole brain areas. Then the resulting FC maps were transformed into Z maps using Fisher's Z transformation.

### 2.9 Statistical analysis

#### 2.9.1 Demographic analyses

IBM SPSS 24 statistical software was used for the statistical analyses. The normal distribution of continuous variables was assessed. Group differences were analyzed using one‐way analysis of variance (ANOVA), *t*‐test or Kruskal–Wallis test by rank (nonparametric) for continuous variables and the Chi‐square test for categorical variables. The significance level was set at *p* < 0.05 for two‐sided tests. Continuous variables are presented as the mean and standard deviation. Categorical variables are shown as absolute and relative frequencies.

#### 2.9.2 Imaging analyses

The DPABI toolbox was used for statistical analyses of imaging data. We performed a mixed‐effect analysis to explore the potential interaction effects between *APOE* (ε2/ε3 carriers vs ε3 homozygotes) and cognitive status (CN and MCI). Second, we explored the main effect of *APOE* ε2 allele and the effect on cognitive status. In addition, age, sex, and education were used as covariates (voxel‐level *p* < 0.001 cluster level *p* < 0.05, Gaussian random field (GRF) correction). Finally, we generated three statistical maps, which include a t‐map showing the effect of *APOE* ε2 allele, a t‐map showing the effect of cognitive state, and an F‐map showing the interaction effect between APOE allele and cognitive state. To further understand how *APOE* and cognitive status interacted to affect regional FC, we also performed post hoc pairwise comparisons after extracting the mean FC value of significant correlations (*p* < 0.05, Bonferroni's correction).

### 2.10 Correlation analyses

We further examined the potential relationships between the imaging metrics, significant FC international effects, and cognitive scores. Additionally, the same analyses were conducted between the average FC and pathological index (Aβ and Tau SUVR). Notably, all analyses were performed among the four groups and for only *APOE* ε2 carriers (CN and MCI). Age, sex, and education level were used as covariates (*p* < 0.05, Bonferroni's correction).

## 3 RESULTS

### 3.1 Demographic and clinical characteristics

The demographic characteristics of all participants are presented in Table 1. There were significant differences among the four groups regarding global cognitive scores (MOCA), memory function, language performance, executive function, language ability, and visual–spatial function. MCI patients had poorer cognitive scores and a greater pathological deposition than those of CN subjects. However, no differences were found in sex, age, or education among the four groups (*p* < 0.05, Bonferroni's correction).

**TABLE 1:** Demographic Statistical results

	APOEε3/ε3 CN	APOE ε2/ε3 CN	APOE ε3/ε3 MCI	APOE ε2/ε3 MCI	*F* (*χ* ^2^)	*p*‐Value
*N*	73	36	60	18		
Age, y	73.05 ± 5.81	73.21 ± 6.83	70.67 ± 6.77	72.31 ± 7.67	1.676	0.174
Sex (F/M)	45/28	20/16	29/32	8/10	1.053	0.370
Education, y	16.67 ± 2.39	16.32 ± 2.62	16.21 ± 2.27	15.50 ± 2.85	1.257	0.291
Global cognitive						
MOCA	26.45 ± 2.46	26.00 ± 2.74	23.36 ± 3.06	23.61 ± 3.82	–	0.009^abc^
Memory						
WMS‐LM immediate recall	15.38 ± 3.15	13.66 ± 3.99	9.95 ± 3.95	10.78 ± 4.01	–	<0.001^abcde^
WMS‐LM delayed recall	14.44 ± 3.58	12.26 ± 3.93	7.98 ± 3.64	9.17 ± 4.74	–	<0.001^abcde^
Attention						
TMT‐A	31.78 ± 10.19	34.78 ± 14.34	40.47 ± 21.09	35.5 ± 9.43	–	0.020^a^
Execution						
TMT‐B	68.59 ± 30.52	69.89 ± 31.14	103.80 ± 64.54	100.39 ± 50.33	–	<0.001^abce^
Language						
BNT	28.75 ± 1.75	28.12 ± 2.09	27.17 ± 2.99	26.54 ± 6.29	–	0.014^a^
Visuospatial function						
CDR	4.78 ± 0.48	4.89 ± 0.32	4.32 ± 0.89	4.44 ± 0.86	–	<0.001^abc^
Aβ Summary SUVR	1.06 ± 0.12	1.07 ± 0.10	1.19 ± 0.29	1.03 ± 0.13	3.994	0.009^d^
Tau Braak I–II SUVR	1.06 ± 0.10	1.08 ± 0.12	1.16 ± 0.19	1.14 ± 0.19	2.538	0.061
Tau Braak III–IV SUVR	1.09 ± 1.08	1.10 ± 0.08	1.19 ± 0.15	1.10 ± 0.13	–	0.004^af^
Tau Braak V–VI SUVR	1.02 ± 0.07	1.03 ± 0.08	1.10 ± 0.08	1.00 ± 0.10	–	0.003^abf^

*Note*: Data are presented as mean ± SD. a–d Post hoc analysis further revealed the source of ANOVA difference (^a^ *APOE* ε3/ε3 CN vs *APOE* ε3/ε3 MCI. ^b^ *APOE* ε3/ε3 MCI vs *APOE* ε2/ε3 CN. ^c^ *APOE* ε2/ε3 CN vs *APOE* ε2/ε3 MCI). ^d^ *APOE* ε3/ε3 CN vs *APOE* ε2/ε3 CN. ^e^ *APOE* ε3/ε3 CN vs *APOE* ε2/ε3 MCI. ^f^ *APOE* ε3/ε3 MCI vs *APOE* ε2/ε3 MCI. (*p* < 0.05, significant difference between the two groups).

Abbreviations: BNT, Boston Naming Test; CDR, Clock Drawing Test; CN, cognitively normal; MCI, mild cognitive impairment; MOCA, Montreal Cognitive Assessment; SUVR, Standard Uptake Value Ratio; TMT, Trail‐making Test; WMS‐LM, Wechsler Memory Scale‐Logical Memory.

### 3.2 FC in BFCS subregions

#### 3.2.1 Disease effects

No significant difference was found for the effect of disease condition (voxel‐level *p* < 0.001, cluster level *p* < 0.05, GRF).

#### 3.2.2 APOE ε2 genotype effects

**FIGURE 1:**
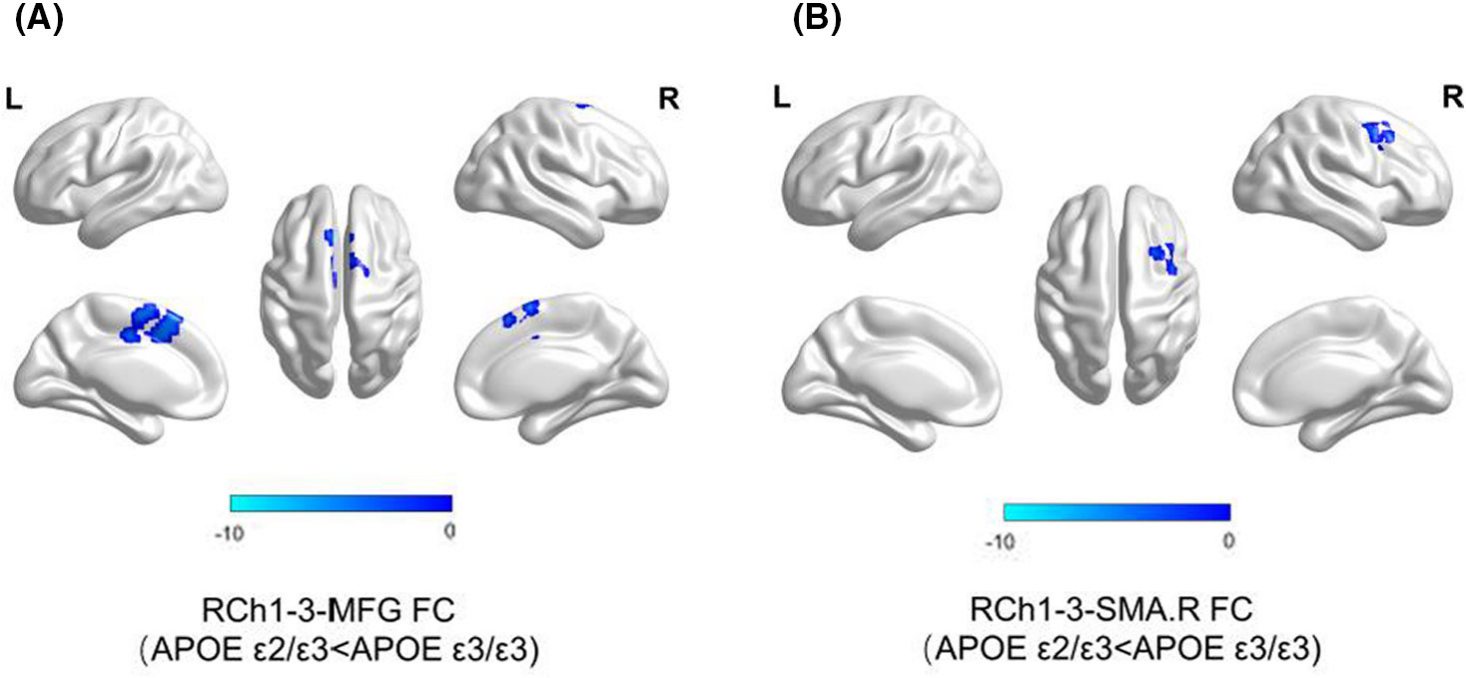
The APOE ε2 genotype effect. (A) The RCh1‐3‐MFG FC strength of *APOE* ε2/ε3 carriers was lower than *APOE* ε3/ε3 carriers. (B) The RCh1‐3‐SMA.R FC strength of *APOE* ε2/ε3 carriers was lower than *APOE* ε3/ε3 carriers. (voxel‐level *p* < 0.001, cluster level *p* < 0.05, Gaussian random field, GRF)

In *APOE* ε2 carriers, decreased connectivity, compared with that of non‐carriers, was found between the following pairs of regions (*p* < 0.05, corrected): (1) right Ch1‐3 and middle frontal gyrus (RCh1‐3‐MFG); (2); right Ch1‐3 and right supplementary motor area (RCh1‐3‐SMA.R). The detailed information is shown in Table 2 and Figure 1.

**TABLE 2:** Mixed‐effect model analysis results across the APOE ε3/ε3 CN, APOE 3/ε3 MCI, APOE ε2/ε3 CN and APOE ε2/ε3 MCI

	Brain region	Peak MNI coordinate			Peak intensity	Number of voxels
		X	Y	Z		
Interaction Effect	RCh4‐LI	−39	−3	9	28	18.18
Interaction Effect	RCh4‐ROL.L	−39	−27	13	13	23.18
genotype Effect	RCh1‐3‐MFG	36	15	36	−4.91	59
genotype Effect	RCh1‐3‐SMA.R	0	12	48	−6.35	122

*Note*: Voxel *p* < 0.001, cluster *p* < 0.05, GRF.

Abbreviations: LI, left Insular; Rol.l, Left Rolandic_Oper; MFG Middle Frontal Gyrus; SMA.R Right Supp_Motor_Area.

#### 3.2.3 Interaction effects of the APOE genotype × disease condition

**FIGURE 2:**
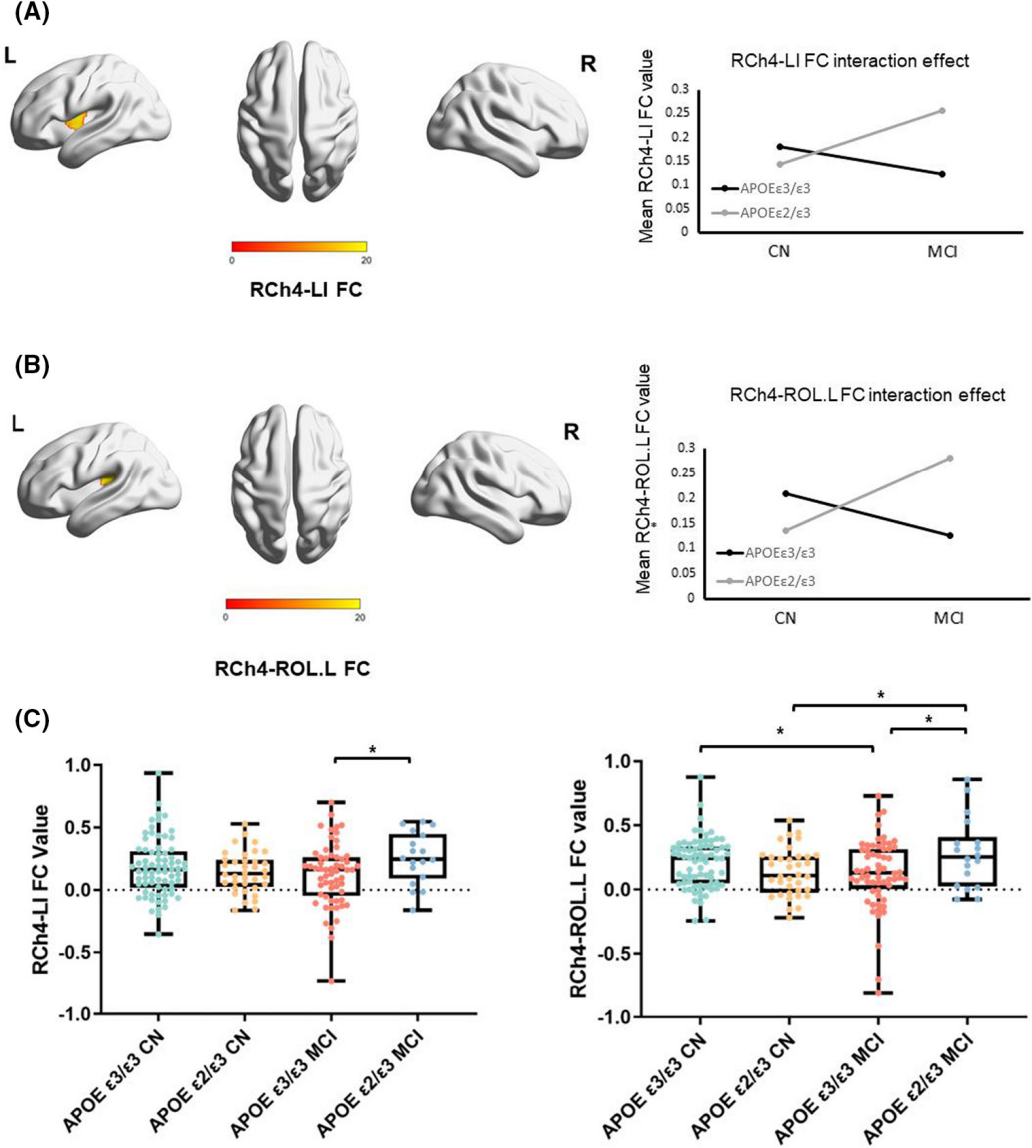
The interaction effect between APOE ε2 allele and cognitive state on functional connectivity. (A) The functional connectivity between RCh4 and Left Insular. (B) The FC between RCh4 and Left Rolandic operculum. (voxel‐level *p* < 0.001, cluster level *p* < 0.05, Gaussian random field, GRF). (C) The mean FC value of RCh4‐LI and RCh4‐ROL.L. *p < 0.05, two‐sample *t*‐test, Bonferroni's correction.

The interaction effects of disease and the *APOE* genotype regarding FC are illustrated in Figure 2 and Table 2. Interaction effects were found between the following pairs of regions (for all analyses, the significance level was *p* < 0.001 at voxel‐level and *p* < 0.05 at cluster level, GRF): initially, right Ch4 and left insula (RCh4‐LI). Secondly, right Ch4 and left Rolandic operculum (RCh4‐ROL.L). For the RCh4‐ROL.L FC, the post hoc analysis illustrated differences among the four groups (*F* = 3.114, *p* = 0.028, Bonferroni's correction). Regarding the RCh4‐LI FC, no significant difference was observed (*F* = 1.868, *p* = 0.137, Bonferroni's correction). Furthermore, MCI subjects with the *APOE* ε2/3 genotype showed the highest connectivity for the RCh4‐LI and RCh4‐ROL.L (Figure 2).

### 3.3 Association between FC and the cognitive scores and pathological changes

**FIGURE 3:**
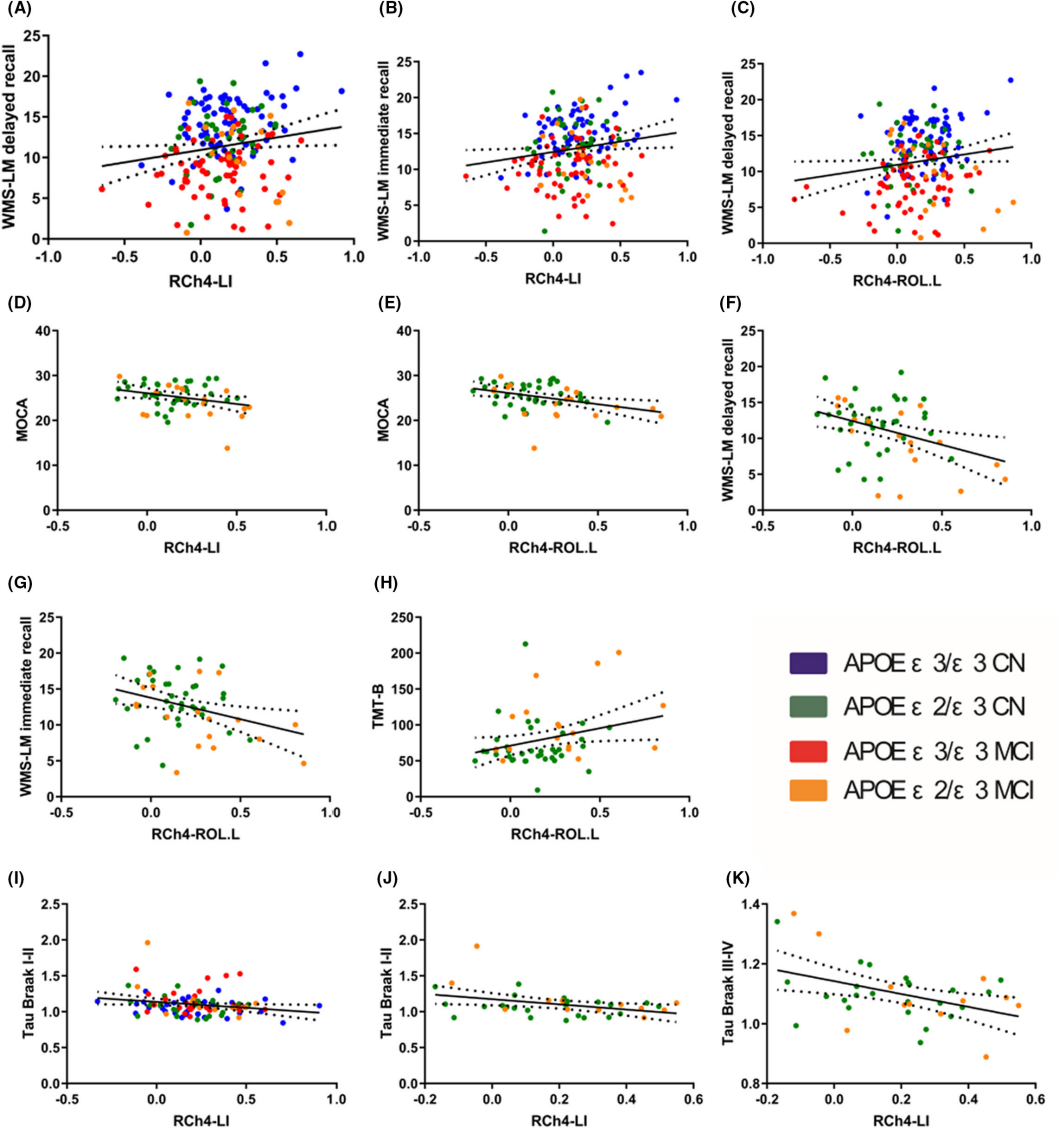
The correlation figures of functional connectivity metrics and cognitive scores as well as pathological burden (partial correlation, age, sex and education were regarded as covariates, *p* < 0.05). Figure (A–D and I) was calculated in all subjects. Figure E‐H and J‐K were calculated in *APOE* ε2 carriers. (A) RCh4‐LI FC strength was positively correlated with WMS‐LM immediate recall (*r* = 0.163, *p* = 0.028). (B) RCh4‐LI FC strength was correlated with WMS‐LM delayed recall (*r* = 0.156, *p* = 0.035). (C) RCh4‐ROL.L FC strength was correlated with WMS‐LM delayed recall (*r* = 0.151, *p* = 0.041). (D) RCh4‐LI FC value was correlated with MOCA (*r* = −0.285, *p* = 0.043). (E) RCh4‐ROL.L FC value was correlated with MOCA (*r* = −0.365, *p* = 0.008). (F) RCh4‐ROL.L FC value was correlated with WMS‐LM delayed recall (*r* = −0.366, *p* = 0.009). (G) RCh4‐ROL.L FC value was correlated with WMS‐LM immediate recall (*r* = −0.347, *p* = 0.014). (H) RCh4‐ROL.L FC was correlated with TMT‐B (*r* = 0.277, *p* = 0.049). (I) RCh4‐LI FC value was correlated with tau Braak I–II SUVR (*r* = −0.216, *p* = 0.034). (J) RCh4‐LI FC value was correlated with tau Braak I–II SUVR (*r* = −0.381, *p* = 0.029). (K) RCh4‐LI FC value was correlated with tau Braak III–IV SUVR (*r* = −0.430, *p* = 0.013).

Regarding the correlations of all subjects, RCh4‐LI FC and RCh4‐ROL.L FC were significantly correlated with memory function (Figure 3, Table 3). However, RCh4‐LI connectivity was negatively associated with the tau Braak stage I‐II SUVR (*r* = −0.216, *p* = 0.034; Figure 3, Table 4). Moreover, higher RCh4‐LI and RCh4‐ROL.L FC was also associated with lower tau Braak III‐IV or V‐VI SUVR values, but these negative correlations were trends (*p* > 0.05, Table 4). The *p*‐values were not significant after correction for multiple comparisons (*p* < 0.05/14 for neuropsychological assessments; *p* < 0.05/8 for pathology analysis).

**TABLE 3:** Correlations between FC metrics and Cognitive scores

	All subjects				*APOE* ε2 carriers			
	RCh4‐LI FC		RCh4‐ROL.L FC		RCh4‐LI FC		RCh4‐ROL.L FC	
	*r*	*p*	*r*	*p*	*r*	*p*	*r*	*p*
MOCA	−0.042	0.569	−0.046	0.536	−0.285	0.043*	−0.365	0.008*
TMT‐A	0.06	0.417	0.092	0.216	0.130	0.364	0.257	0.068
TMT‐B	−0.053	0.473	−0.025	0.732	0.198	0.164	0.277	0.049*
WMS‐LM immediate recall	0.163	0.028*	0.127	0.086	−0.172	0.231	−0.347	0.014*
WMS‐LM delayed recall	0.156	0.035*	0.151	0.041*	−0.161	0.265	−0.366	0.009*
BNT	0.133	0.127	0.095	0.278	−0.076	0.707	−0.067	0.739
CDR	−0.050	0.503	−0.118	0.110	−0.246	0.082	−0.248	0.079

*Note*: No significant *p*‐value after multiple comparison correction (*p* < 0.05/14).

Abbreviations: LI, left Insular; Rol.l, Left Rolandic_Operculum; MFG, Middle Frontal Gyrus; SMA.R Right, Supp_Motor_Area; FC, Functional Connectivity; MOCA, Montreal Cognitive Assessment; WMS‐LM, Wechsler Memory Scale‐Logical Memory; TMT, Trail‐making Test; BNT, Boston Naming Test; CDR, Clock Drawing Test.

* *p* < 0.05, uncorrected.

**TABLE 4:** Correlations between FC metrics and pathological burden

	All subjects				*APOE* ε2 carriers			
	RCh4‐LI FC		RCh4‐ROL.L FC		RCh4‐LI FC		RCh4‐ROL.L FC	
	*r*	*p*	*r*	*p*	*r*	*p*	*r*	*p*
Tau Braak I–II SUVR	−0.216	0.034*	−0.041	0.688	−0.381	0.029*	0.075	0.680
Tau Braak III–IV SUVR	−0.135	0.188	−0.032	0.756	−0.430	0.013*	−0.095	0.598
Tau Braak V–VI SUVR	−0.098	0.340	−0.025	0.811	−0.335	0.057	−0.129	0.473
Aβ SUVR	0.015	0.867	0.022	0.808	−0.083	0.634	0.005	0.978

*Note*: No significant *p*‐value after multiple comparison correction (*p* < 0.05/8).

Abbreviations: LI, left Insular; Rol.l, Left Rolandic_Operculum; FC: Functional Connectivity.

* *p* < 0.05, uncorrected.

Among *APOE* ε2 carriers, those with higher RCh4‐LI FC exhibited poorer MOCA scores, memory function and executive function (Figure 3, Table 3). This negative association was also observed for pathological findings (Figure 3, Table 4). Specifically, negative relations were detected between RCh‐L.LI FC and tau Braak stage I–II (*r* = −0.381, *p* = 0.029) and III‐IV (*r* = −0.43, *p* = 0.013). Notably, no correlation was found between imaging metrics and Aβ pathology among all subjects or ε2 carriers alone. The *p*‐values were not significant after correction for multiple comparisons (*p* < 0.05/14 for correlation analyses of neuropsychological assessments; *p* < 0.05/8 for correlation analyses of pathology analysis).

## 4 DISCUSSION

The current study investigated the interaction effect between the *APOE* ε2 genotype and cognitive status based on the FC of BFCS subregions. Our main findings were as follows: (1) interaction effects between the *APOE* ε2 genotype and cognitive status was found for the LI and ROL.L regions, (2) alteration of FC patterns of BFCS subregions were also associated with general cognitive function and tau pathology, and (3) *APOE* ε2 carriers showed decreased FC of the RCh1‐3 to several cortical regions including the MFG and SMA.R. Our study might provide original insights for understanding the possible protective role of *APOE* ε2 alleles in MCI patients through the cholinergic pathway.

### 4.1 Effect of the APOE ε2 genotype

*APOE* ε2 carriers, regardless of disease condition, showed decreased FC between the right Ch1‐3 and frontal regions involving the MFG and SMA.R compared with that in non‐carriers. Notably, cholinergic neurons of the BFCS have been shown to project to the frontal cortex.^42^, ^43^ Additionally, upregulation of choline acetyltransferase (ChAT) activity in the frontal cortex in MCI patients may be related to BFCS neurons, which innervate the frontal cortex.^44^ As a part of the frontal lobe, the MFG region is closely associated with working memory and executive function,^45^ and the pathological damage to the MFG is vital in the AD spectrum.^46^, ^47^, ^48^ Additionally, MCI patients showed decreased FC between the MFG and BFCS regions.^24^ Moreover, the SMA occupies the posterior one‐third of the superior frontal gyrus. Disconnection of the tract to this area is also relevant for cognitive function, especially for those in the early stages of AD requiring constructional praxis.^49^, ^50^ Furthermore, the abnormal pattern of FC between the BFCS and SMA was correlated with cognitive performance.^24^ Herein, we report the decreased FC of the BFCS in *APOE* ε2 carriers. The possible explanation is that the protective effect of the ε2 alleles is related to decreased neural activity.^51^ Specifically, some studies have indicated that elderly ε2 carriers show reduced long‐term potentiation (LTP) activity, which results in diminished Aβ excitotoxicity, and thus promotes neuroprotection.^52^ Accordingly, our finding regarding the reduction of BFCS FC adds evidence for understanding the effect of the *APOE* ε2 allele on the brain. It may account for the correlation of the ε2 allele with decreased AD susceptibility.

### 4.2 Interactive effect between APOE ε2 and cognitive status

Our study found an interactive influence between clinical status and the *APOE* genotype on BFCS FC between the RCh4 and LI and RCh4 and ROL.L. MCI patients with the *APOE* ε2 allele had the highest average RCh4‐LI and RCh4‐ROL.L FC (Figure 2). The central sulcus of the insula is the most inferior extension of the Rolandic fissure, and the insular lobe has extensive FC to the ROL.^53^ These areas are also linked to cognition performance.^54^, ^55^ Previous studies have revealed that the insula is affected in MCI,^56^ and insular degeneration at risk for developing AD.^57^ Furthermore, the intermediate Ch4 subdivision provides the primary cholinergic input for the insula and temporal area.^43^ Some studies have found decreased FC of the Ch4 region to the insula in MCI patients.^23^, ^24^ However, other studies have showed opposite outcomes. *APOE* ε2 carriers had increased FC in subnetworks of the default mode network (DMN) subnetwork,^58^ bilateral middle temporal gyrus (MTG), right precuneus (PCUN) and right precentral gyrus (PreCG) than *APOE* ε3 homozygous carriers.^59^ Moreover, another interesting finding of this study is that *APOE* ε2 carriers with MCI presented increases in BFCS FC patterns, which may suggest a possible protective effect of the *APOE* ε2 allele. Previous studies have consistently indicated protective clinical and pathological roles of *APOE* ε2, such as delayed emergence of AD,^8^ decreased Aβ and tau deposition in the brain,^35^, ^60^ increased gray matter volume,^61^ and slower cognitive decline.^62^ Furthermore, our previous study has also shown increased brain activity in MCI patients carrying the *APOE* ε2,^63^ suggesting a potential compensatory mechanism in MCI.^59^, ^64^

Interestingly, our study reported decreased BFCS FC in CN *APOE* ε2 carriers compared with CN *APOE* ε3 homozygotes. Some studies have also demonstrated that *APOE* ε2 carriers showed decreased FC compared with homozygous ε3 carries among older CN subjects.^65^ Various *APOE* genotypes may differently influence disease development stages.^58^ In addition, because Aβ production could be regulated by aberrant network activity,^66^ we speculate that the decrease in FC of ε2 carriers' early life may slow pathological deposition; thereby, increased FC in the later stage could be a protective factor.^65^

### 4.3 Increased BFCS FC was correlated with better cognitive function and decreased tau deposition

Meanwhile, we observed correlations between the FC strength of RCh4‐LI and RCh4‐ROL.L and cognition (Figure 3). Specifically, the FC strength was positively correlated with memory function in all subjects. These findings may reflect that the increased FC strength may be related to improved memory performance. Furthermore, in the group carrying the *APOE* ε2 allele, MCI patients had higher mean FC strength and lower mean MOCA and memory function scores than those of CN subjects. However, the RCh4‐ROL.L FC value was positively associated with executive function. Moreover, the demographic analysis revealed no significant difference between CN subjects and MCI patients carrying the *APOE* ε2 allele with regard to memory function, executive function, and MOCA scores. However, MCI patients with the ε2/ε3 genotype had better scores on these evaluations than those of CN subjects with this genotype. Additionally, the *APOE* ε2 allele has been associated with decreased cognitive decline during aging.^67^ On the basis of these findings, we suggest that during the MCI stage, *APOE* ε2 carriers have increased FC, which maintains their cognitive status. Moreover, the mean scores of ε3 homozygotes with MCI were lower than that of CN ε3 homozygotes. Accumulated evidence has confirmed that the MCI stage is related to greater pathological deposition and worse cognition than that of CN subjects,^68^, ^69^ which is supported by our results.

The other novel finding in this study is that FC patterns of the BFCS to the other cortical areas showed significant negative correlations with the pathological burden in all subjects and *APOE* ε2 carriers. Multiple studies have shown that the cholinergic system of BFCS degenerates early in MCI and AD patients because of its vulnerability to tau pathology.^9^, ^70^, ^71^, ^72^, ^73^ Moreover, recent studies have indicated that the link between tau pathology and cholinergic deficits might be initiated early during aging‐MCI‐AD.^70^, ^73^, ^74^ Also, some researchers have found that ε2 was independently associated with a lower Braak NFT stage.^35^ The findings of our current study support those findings. The FC changes of the BFCS were related to cortical pathology, especially the tau burden, in all subjects and *APOE* ε2 carriers. *APOE* ε2 carriers in the MCI stage displayed higher RCh4‐LI and RCh4‐ROL.L FC concerning the interaction effects and were associated with decreased tau deposition in the entorhinal‐perirhinal cortex and hippocampus. These regions are vulnerable to tau spread in individuals with a limbic‐predominant AD phenotype according to one data‐driven model.^75^ This observation implies a potentially protective role of the *APOE* ε2 allele against the deposition of NFTs in the BFCS and their spread to the cerebral cortex before pathological progression.

There are some limitations to the current study that should be noted. First, the sample size of *APOE* ε2 carriers with MCI was relatively small compared with that of the other three groups. Because *APOE* ε2 homozygotes comprise <1% of the general population,^6^, ^76^ it is challenging to recruit these subjects. Thus, caution should be applied when interpreting the compensatory mechanism mediated by *APOE* ε2 because of the small sample size. Further studies enrolling additional subjects are needed to confirm the effect of APOE ε2. Second, some individuals did not have AV‐45 or AV‐1451 PET data, which reduced the statistical power. Finally, increasing evidence shows that genetic variations play a crucial role in sporadic AD. Aside from APOE allele being the most acknowledged factor, genome wide association studies (GWAS) have identified over 50 genetic factors associated with AD, such as NGFR, TREM2, CD33, ABCA7, MS4A6A, and CD2AP.^77^, ^78^ It is necessary to further validate the relationship between genetic polymorphism with the disease. Besides, multiple factors lead the onset of sporadic AD and there are several exploratory topics of future research. The vascular etiology is an important part of the disease process,^79^ such as the hypoperfusion and dysfunction of blood brain barrier.^80^ Additionally, visual and auditory impairments have been found in MCI or AD patients and correlate with cognitive function.^81^, ^82^ Moreover, it is clear that the gut‐brain axis plays a vital role in neurodegenerative diseases. And constipation has been considered to be a factor of cognitive decline in AD and MCI patients.^83^ Future studies need to be conducted to explore more details.

In conclusion, the results of our study provide evidence that the *APOE* ε2 allele impacts the FC patterns of BFCS subregions in MCI patients, which may be an important mechanism contributing to its protective role. Furthermore, the *APOE* ε2 allele is associated with decreased tau deposition in the BFCS subregions regardless of cognitive status.

## AUTHOR CONTRIBUTIONS

Xiaocao Liu and Qingze Zeng designed the study. Xiaocao Liu wrote the first draft of the manuscript. Xiaocao Liu and Qingze Zeng collected the clinical and MRI data. Xiao Luo analyzed the MRI data and wrote the protocol. Kaicheng Li, Luwei Hong, Jixuan Li, Xiaojun Guan, Xiaojun Xu, Peiyu Huang, and Minming Zhang assisted with the research design and interpretation of the results. Xiaopei Xu had polish the article. All authors contributed to the final manuscript. And all authors contributed to read, and approved the final manuscript.

## CONFLICT OF INTEREST

All authors report no financial interests or potential conflicts of interest.

## INFORMED CONSENT

Written informed consent was obtained from all participants, authorized representatives, and study partners before any protocol‐specific procedures were carried out in the ADNI study.

## Supporting information


Figure S1.

## Data Availability

The datasets generated and/or analyzed during the current study are available in the ADNI study. Additional details can be found in http://www.adni‐info.org.
