# Supplementary material for: Effects of APOE ε2 allele on basal forebrain functional connectivity in mild cognitive impairment
Source: CNS Neurosci Ther. 2022 Dec 5;29(2):597–608. doi: 10.1111/cns.14038 (PMC9873529; doi:10.1111/cns.14038)

**Supplementary Materials**

**Figure S1** The flow diagram of the participants selection and statistical analysis.


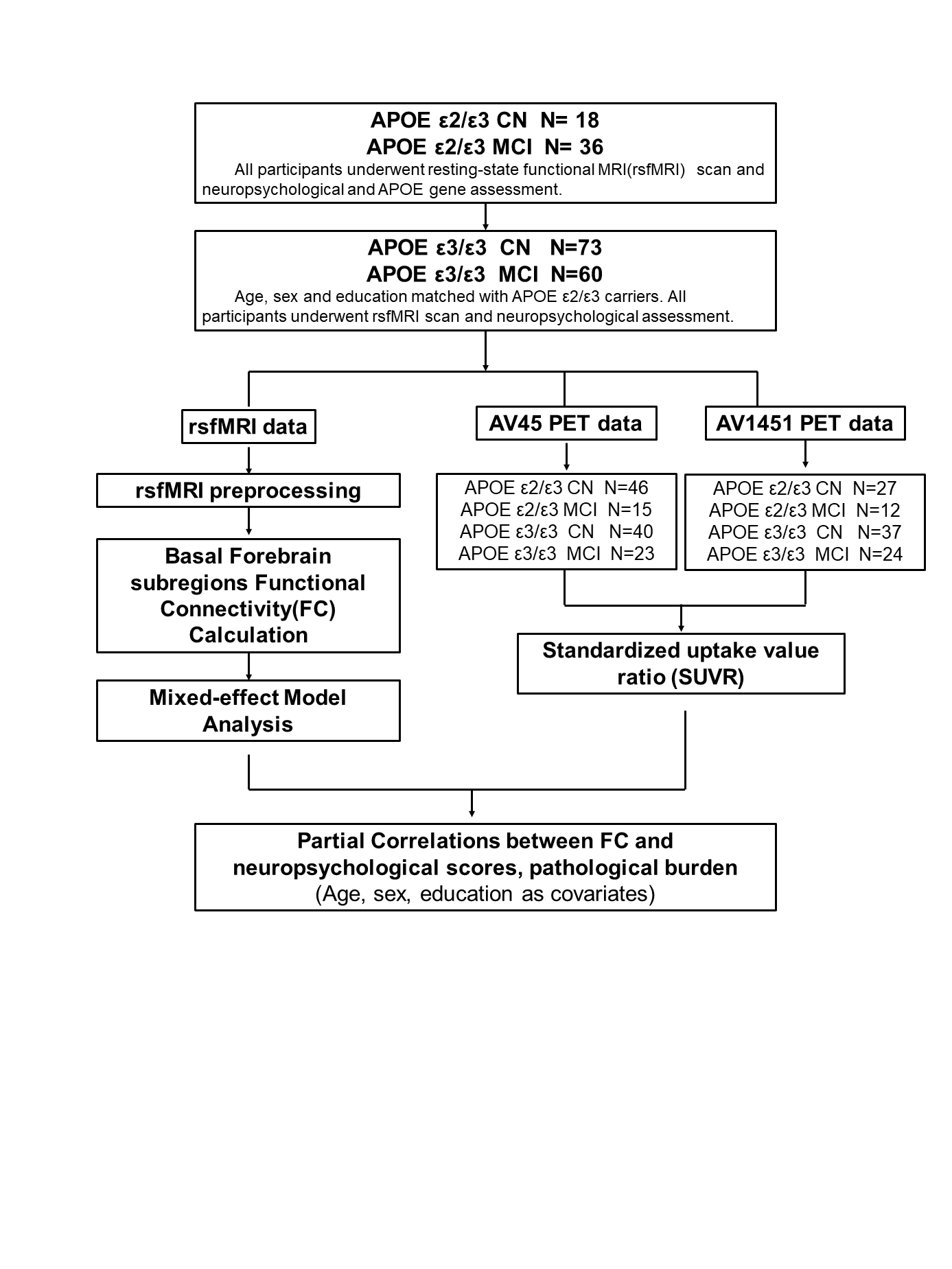


Abbreviation: *CN,* Cognitively Normal; *MCI,* Mild Cognitive Impairment

**Figure S2** Schematic diagram of basal forebrain seeds


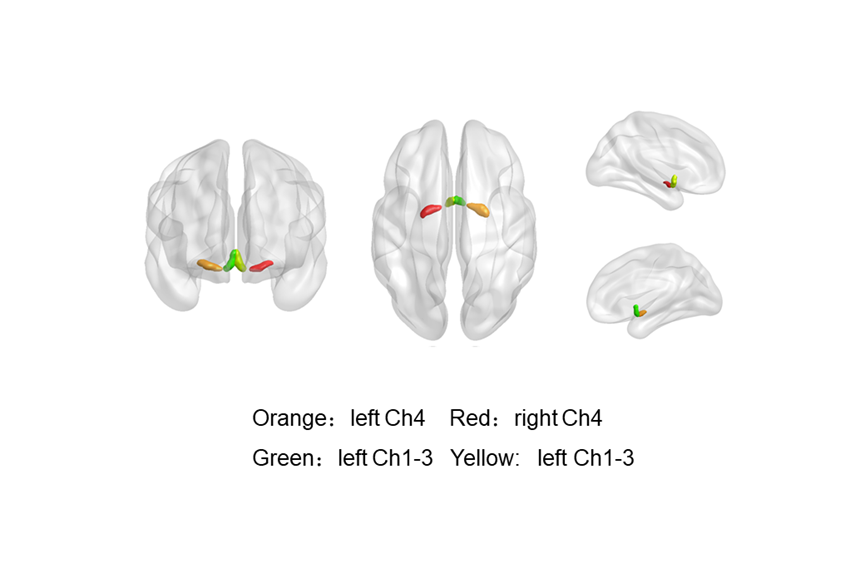

Supplement: Supplementary file 1 — Figure S1. [file CNS-29-597-s001.docx]
